# Development and growth of organs in living whole embryo and larval grafts in zebrafish

**DOI:** 10.1038/s41598-017-16642-5

**Published:** 2017-11-28

**Authors:** Toshihiro Kawasaki, Akiteru Maeno, Toshihiko Shiroishi, Noriyoshi Sakai

**Affiliations:** 1 Genetic Strains Research Center, National Institute of Genetics, Mishima, 411-8540 Japan; 2 Department of Genetics, School of Life Science, SOKENDAI (The Graduate University for Advanced Studies), Mishima, 411-8540 Japan; 3 Technical Section, National Institute of Genetics, Mishima, 411-8540 Japan

## Abstract

Age-related systemic environments influence neurogenesis and organ regeneration of heterochronic parabiotic partners; however, the difficulty of manipulating small embryos prevents the effects of aged systemic environments on primitive organs at the developmental stage from being analysed. Here, we describe a novel transplantation system to support whole living embryos/larvae as grafts in immunodeficient zebrafish by the intrusion of host blood vessels into the grafts, allowing bodies similar to those of heterochronic parabiosis to be generated by subcutaneous grafting. Although grafted embryos/larvae formed most organs, not all organogenesis was supported equally; although the brain, eyes and the intestine usually developed, the liver, testes and heart developed insufficiently or even occasionally disappeared. Removal of host germ cells stimulated testis development in grafted embryos. These results indicate that primitive testes are susceptible to the systemic environments that originated from the germ cells of aged hosts and imply that the primitive liver and heart are similar. Upon applying this method to embryonic lethal mutants, various types of organs, including testes that developed in germ-cell-removed recipients, and viable offspring were obtained from the mutants. This unique transplantation system will lead to new insights into the age-related systemic environments that are crucial for organogenesis in vertebrates.

## Introduction

Parabiotic pairings, which share a circulatory system by surgical connections between two entire animals, show unique phenomena of rescuing individuals that were lethally damaged by radiation^1^ and affecting organ growth^2,3^. Recently, heterochronic parabiosis in mice has advanced in ageing studies because age-related systemic environments influence neurogenesis and organ regeneration in heterochronic partners^4–6^. However, a rejection response and the difficulty of manipulating the connection between two individual animals likely prevents the production of heterochronic parabiotic pairs in most vertebrates. In the sexual parasitism of deep-sea ceratioid anglerfishes, the male is joined to the female on the belly, and attachment is followed by the fusion of epidermal tissues and, eventually, the uniting of the circulatory systems^7^. Syngnathus pipefish males care for their embryos in a brood pouch, where paternally derived blood vessels contact the embryos^8^. These unique properties of fish epidermal tissues hint at an alternative approach to generate parabiosis in teleosts.

We recently established a subcutaneous transplantation system of the adult testis using immunodeficient *rag1* mutant zebrafish, in which the immune system does not reject allogeneic tissue^9^. The grafted fragment of testicular hyperplasia grows subcutaneously, and host blood vessels intrude into the grafts. Since the size of zebrafish embryos and larvae is smaller than the size of the fragment, it is possible to graft the whole-body subcutaneously, allowing us to transplant all of the tiny organs in the body simultaneously. Therefore, we sought to establish a transplantation system for living whole embryos and larvae to achieve whole-organism transplantation subcutaneously in adult zebrafish. The results show that transplantation of a living whole embryo or larva into *rag1* mutant recipients can support development, including of embryonic lethal mutants, by the intrusion of host blood vessels into the grafts. Although primitive organs such as the liver, heart and testes in the grafted embryos occasionally disappeared 2 months after transplantation, germ cell-removed hosts recovered the support for testis development in grafted embryos, giving rise to the possibility that age-related systemic environments influence the development of primitive organs such as the liver, heart and testis in zebrafish.

## Results

### Growth of embryos after primitive body structure formation by subcutaneous grafting

**Figure 1:**
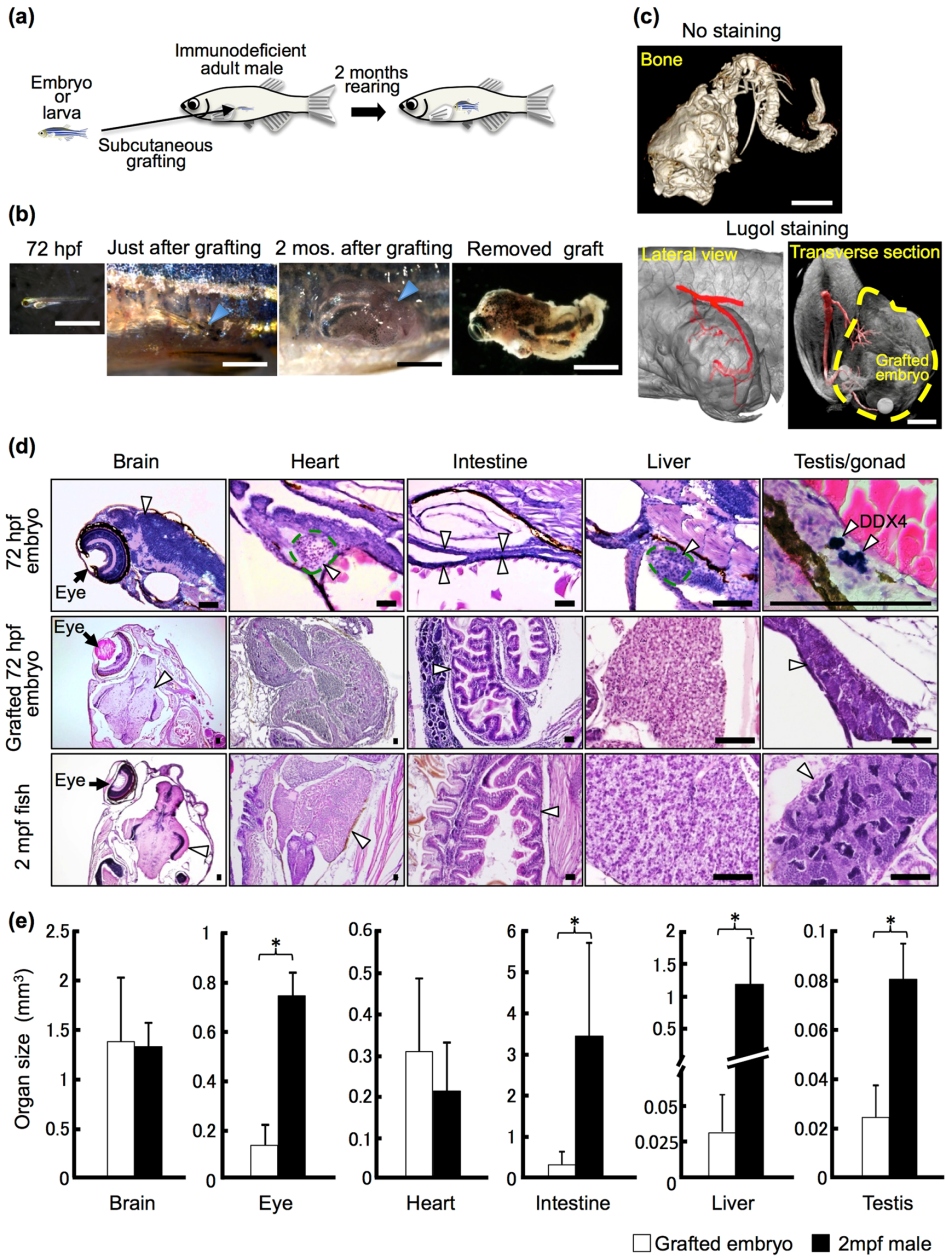
Development of grafted 72 hpf embryos and organ growth in *rag1* mutant hosts. (**a**) Schematic of whole-embryo transplantation. (**b**) Representative 72 hpf embryos and grafted embryos. Arrowheads indicate the site of the subcutaneous embryo graft. Scale bar: 2 mm. (**c**) µ-CT imaging of the grafted embryo 2 months after transplantation. Cranial bones and vertebrae were observed in the grafted embryos without staining. Using Lugol staining, a vascular structure was observed. Note that the posterior cardinal vein (red) of the recipient is connected to the grafted embryo (yellow dotted line). Scale bar: 1 mm. (**d**) Histology of the 72 hpf embryo, the 72 hpf embryo graft 2 months after transplantation, and the 2 mpf fish. Germ cells of 72 hpf embryos were stained with anti-DDX4 (Vasa) antibody to identify the primitive gonad. Arrowheads indicate each organ as labelled above the panels. Scale bar: 80 µm. (**e**) Comparison of organ size between grafted 72 hpf embryos 2 months after transplantation and normal 2 mpf males. The volume of each organ was calculated from serial sections of grafts (n = 12 brains, 11 eyes, 11 hearts, 12 intestines, 8 livers, and 6 testes) and of 2 mpf males (n = 3). Error bars indicate standard deviation. *p < 0.01 (Student’s t-test).

First, we performed subcutaneous transplantation of embryos at 72 hours post-fertilization (hpf), just after they had hatched and completed most morphogenesis. The grafted embryos were maintained and grown in the *rag1*−/− recipients, and their shape was relatively similar to that of fish at 2 months post-grafting (Fig. 1a,b). A beating heart was observed in these grafted embryos (Supplementary Video S1).

**Figure 2:**
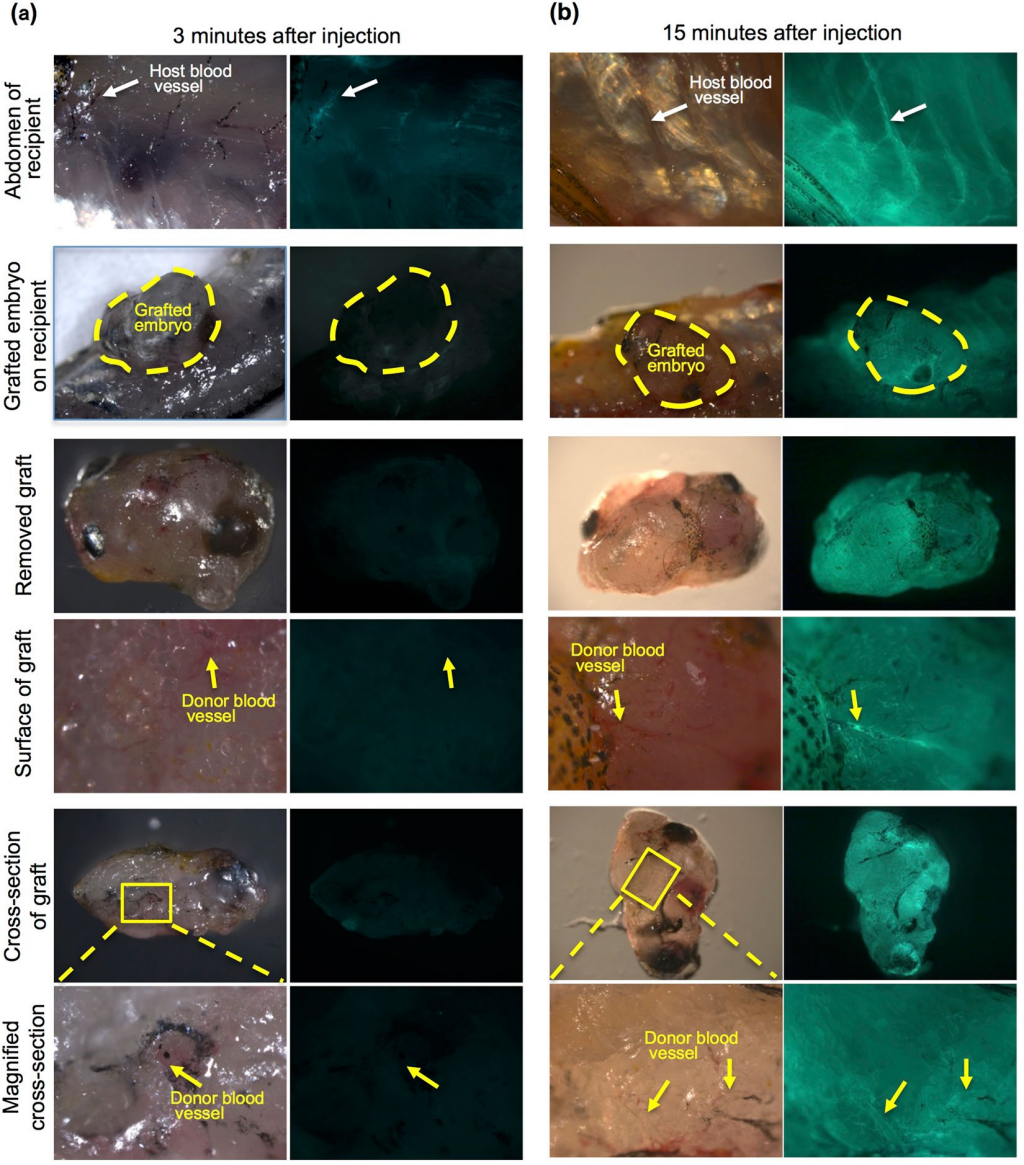
Microinjection of FITC-dextran into the recipient heart. To examine vascularization between recipients and grafted embryos, FITC-conjugated dextran solution was injected into the hearts of living recipients in which 72 hpf embryos had been transplanted 3 months previously. (**a**) At 3 minutes after injection, slight fluorescence of the recipient blood vessels (white arrow) was observed in the recipient abdomen but not in grafted embryos (yellow dotted line). No fluorescence in the blood vessels was observed in the grafted embryo (yellow arrows). The recipient heart in which FITC-dextran was injected is outside the frame of this panel. Most of the FITC-dextran has not reached the abdomen at 3 minutes after injection. (**b**) At 15 minutes after injection, strong fluorescence was observed in the recipient blood vessels (white arrow) of the abdomen and the grafted embryos (yellow dotted line). This strong fluorescence was also observed in the blood vessels of the grafted embryo (yellow arrows). These results suggest that recipient blood vessels are connected with grafted embryos.

Analyses of X-ray µ-CT for grafted 72 hpf embryos showed cranial bones and vertebrae at 2 months post-grafting (Fig. 1c, Supplementary Video S2), suggesting that osteogenesis also proceeded in grafted embryos. When recipients were stained with Lugol’s solution, vascularization that extended from the recipient to the donor embryo was observed (Fig. 1c). In addition, when FITC-labelled dextran solution was injected into recipient hearts, we confirmed the arrival of FITC-labelled dextran into grafted embryos (Fig. 2). These results suggest that the vasculature of grafted embryos connects to the host by sharing blood circulation. This feature likely supports the development of grafted embryos for 2 months.

### Subcutaneous grafting perturbs the normal development of blastula and gastrula embryos

Since the embryos at 72 hpf were maintained and developed for 2 months after subcutaneous transplantation, we next examined the subcutaneous transplantation of embryos at different developmental stages. Whole embryos at 3 hpf (blastula, 1000-cell stage), 6 hpf (early gastrula, shield stage), 24 hpf (completion of somatogenesis) and larvae at 5 days post-fertilization (dpf) were transplanted into *rag1*−/− recipients subcutaneously. The grafted embryos at 24 hpf and larvae at 5 dpf were maintained and developed the same as did embryos that were grafted at 72 hpf (Supplementary Fig. S1a,b). These results indicate that the development of living embryos after the completion of somatogenesis is supported by the subcutaneous condition.

**Figure 3:**
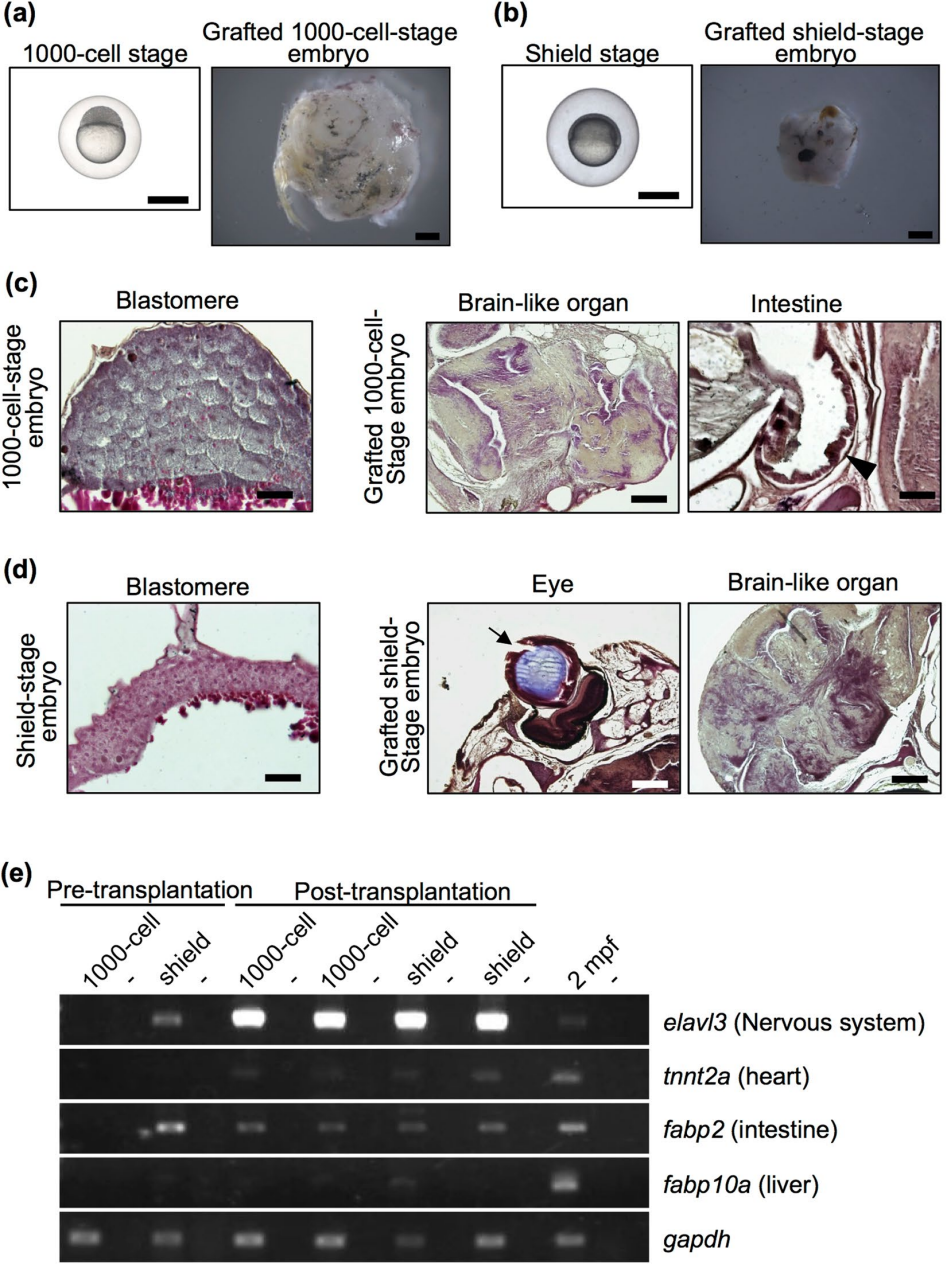
Failure of organ formation in grafted blastular and gastrular embryos. (**a**,**b**) Representative 1000-cell-stage (3 hpf) embryos (**a**), shield-stage (6 hpf) embryos (**b**) and grafted embryos two months after transplantation. Both embryo stages were subcutaneously transplanted into *rag1* mutants. Scale bar: 1 mm. (**c**,**d**) Histology of 1000-cell-stage embryos (**c**) and shield-stage embryos (**d**) before and after transplantation. Sections were stained with haematoxylin and eosin. Although brain-like organs with an irregular shape and cell body arrangement were observed in all of the grafted early-stage embryos, other organs were not observed, except an eye (arrow) and intestine (arrow head) in a few grafted embryos. Scale bars: 200 µm (brain-like organ and eye), 100 µm (intestine), 50 µm (blastula and gastrula). (**e**) RT-PCR analysis of organ-specific genes in the grafted blastular and gastrular embryos at 2 months post-transplantation. Each experiment was conducted using two independent samples. Wild-type zebrafish at 2 mpf were used as positive controls. Negative controls lacking reverse transcriptase are located in alternating lanes (−). *gapdh* was used as a positive control.

In contrast, although almost all of the grafted embryos at the 1000-cell (3 hpf) and shield stages (6 hpf) were maintained 2 months after transplantation and their sizes increased, the shapes of the grafted embryos were not similar to that of the normal fish body (Fig. 3a,b). Histological observation did not show most of the organs, except for the brain-like tissue (Table 1; Fig. 3c,d). The intestine and an eye were rarely observed (they were observed in 1 of the 8 samples). To confirm these findings, the expression of the brain-specific marker *elavl3* ^10^, the heart-specific marker *tnnt2a* ^11^, the intestine-specific marker *fabp2* ^12^ and the liver-specific marker *fabp10a* ^13^ was measured by RT-PCR. This analysis indicated the strong expression of the brain-specific marker *elavl3* and the weak expression of the intestine-specific marker *fabp2*, whereas specific markers of the heart, *tnnt2a*, and liver, *fabp10a*, were barely detectable (Fig. 3e).

**Table 1:** Growth of grafted embryos/larvae and development of their organs.

grafted embryo	recipient	# of grafts	# of survived recipients	# of grown grafts	# of grafts with testis formation	# of grafts containing brain	# of grafts containing eye	# of grafts containing heart	# of grafts containing intestine	# of grafts containing liver
3 hpf	*rag1*	13	13*	12	0/8^†^	8/8	0/8	0/8	1/8	0/8
6 hpf	*rag1*	12	12	11	0/4	4/4	1/4	0/4	0/4	0/4
24 hpf	*rag1*	21	20	16	4/9	9/9	8/9	1/9	8/9	3/9
72 hpf	*rag1*	28	21	20	6/14	14/14	14/14	11/14	14/14	9/14
5 dpf	*rag1*	12	12	12	5/7	7/7	7/7	6/7	5/7	3/7
72 hpf	Germ cell-depleted *rag1*	28	27	25	9/10	10/10	9/10	7/10	10/10	7/10
72 hpf	Castrated *rag1*	17	6^§^	6	5/6	6/6	6/6	6/6	6/6	3/6
72 hpf *spns2* mutant	Germ cell-depleted *rag1*	21	15	13	4/8	8/8	8/8	0/8	8/8	6/8
7 dpf *oep* mutant	Germ cell-depleted *rag1*	14	14	14	4/7	7/7	5/7	0/7	1/7	0/7

*The number of recipients that survived for 2 months after transplantation. ^†^The denominator shows the number of grafts used for histological observation; the remaining grafts were used for other analyses. ^§^Because the castrated *rag1* recipients tended to die immediately after transplantation due to damage incurred from castration surgery, only some of the recipients survived for 2 months after transplantation.

Because a hypertonic environment disrupts normal embryogenesis in zebrafish^14^, we examined whether an isotonic environment would affect embryogenesis. When we kept 1000-cell-stage embryos in isotonic medium (300 mOsm/L: a common vertebrate extracellular tonicity), most of the embryos developed normally (Supplementary Table S1). These results suggest that the subcutaneous condition of adult hosts does not support the development of blastulae and early gastrulae, which seem to induce differentiation to mostly ectodermal derivatives.

### Heterogeneous development of organs in grafted embryos and larvae after primitive organ formation

Since subcutaneous conditions in adult hosts do not support the normal development of blastula and gastrula embryos, we performed histological observations of sections after their removal from the host to examine if organogenesis occurred appropriately in the grafted embryos/larvae at 24 hpf, 72 hpf, and 5 dpf. The sections showed that each organ, such as the brain, eyes, heart, intestine, liver, and testes, developed in different ways. The brain, eyes, and intestine developed in most grafted embryos/larvae, whereas the liver, heart and testes were occasionally absent (Table 1; Fig. 1d; Supplementary Fig. S1c). Development of the heart was rarely observed in the grafts of 24 hpf embryos, whereas such development was observed in most grafts at 5 dpf. By RT-PCR analysis of organ-specific genes, the expression of *elavl3* and *fabp2* was detected in all of the grafted embryos and larvae, *tnnt2a* was not expressed in any grafted 24 hpf embryos and *fabp10a* was not observed in some grafted embryos (Supplementary Fig. S1d), thus supporting our histological observations of grafted embryos/larvae. These results indicate that several primitive organs, especially the heart, liver and testes, do not develop under subcutaneous conditions, even though the grafted embryos become larger. They may thus have a period in which they are susceptible to the systemic environments of the adult host and can be severely damaged.

For the size of the organs, we measured the area of each organ in serial sections of grafted 72 hpf embryos 2 months after transplantation. Although the volume of each organ type in 72 hpf embryos increased after transplantation (Supplementary Fig. S2), the size of the liver and testis was smaller than that of normally developing individuals, even in the cases when they developed (Fig. 1e). The development of the eyes and intestine of the subcutaneously grafted 72 hpf embryos also showed changes in size, but the brain developed as same as observed in normal embryos. Although hearts were lost in many grafted embryos at 24 dpf, in grafted embryos at 72 dpf, they developed to almost the same size observed in normal individuals in the cases in which they developed. These results suggest that the development of various primitive organs such as the liver, testes, heart, eyes and intestine are affected by the subcutaneous conditions of adult hosts, although the degree is different for each organ type.

### Removal of germ cells from hosts stimulates testis development in grafted embryos

Testicular function is not necessary for the survival of the individual, and castrated mouse hosts are used for grafting testis tissue of newborn mice, pigs or goats^15^. Similarly, germ cells can be removed from zebrafish via knock down of *dead end* using morpholino antisense oligonucleotides^16^. Therefore, we examined testis development in 72 hpf embryos grafted into castrated and germ-cell-depleted *rag1* mutants to determine which cells of the host testis affect the growth of primitive gonads in grafted embryos.

**Figure 4:**
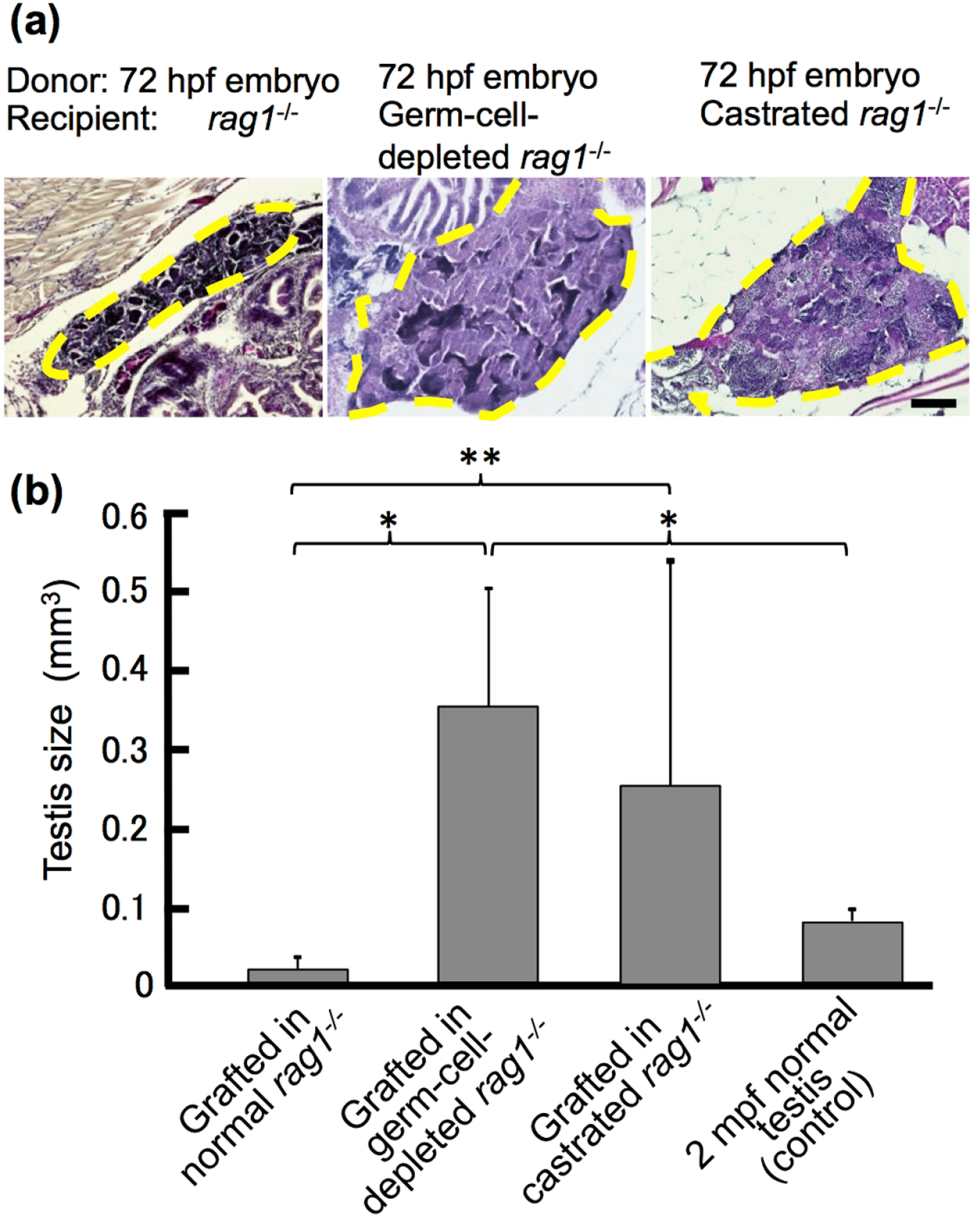
Growth of testis in 72 hpf embryos grafted into germ cell-depleted and castrated *rag1* mutants after 2 months. (**a**) Histology of testes of 72 hpf embryos grafted into normal, germ cell-depleted, and castrated *rag1* mutants. Dotted lines represent testes of the grafts. Scale bar, 100 µm. (**b**) Comparison of testis size between 2 mpf normal testes and grafts into normal, germ cell-depleted and castrated *rag1* mutants. Testes volume was calculated from graft serial sections (n = 9 normal, n = 7 germ cell-depleted and n = 5 castrated) and of 2 mpf males (n = 3). Error bars indicate the standard error. *p < 0.01; **P < 0.05 (paired t-test).

At two months after transplantation, the gonads of 72 hpf embryo grafts in either the castrated or germ-cell-depleted hosts developed more frequently than did those in embryo grafts in the normal host (Table 1). The donor testes in the germ-cell-depleted host became approximately 30 times larger than those in the normal host and 4 times larger than the testes of normal fish at 2 months post-fertilization (mpf) (Fig. 4). The size of the donor testis in the castrated host was also larger than that in the normal host and varied widely. Since we observed a small fragment of the testis remaining in the host in which testes of the grafted embryo did not develop well at 2 months after transplantation, the wide variation in castrated hosts is probably due to the incomplete removal of host testes. Spermatogenesis was confirmed by artificial insemination using well-developed testes of the graft at 2 months post-grafting, and the resultant fertilized eggs developed normally (Supplementary Table S2). These results indicate that the host germ cells are the origin of the systemic environment that prevents testis development in the grafted embryo.

### Generation of sperm from embryonic lethal mutants

Since germ-cell-depleted recipients stimulated constant testis growth in grafted embryos, we asked if this method supported spermatogenesis in embryonic lethal mutants, such as those with severe defects in the heart, which prevents the analysis of spermatogenesis beyond the lethal stage. Therefore, we examined the possibility of sperm generation in two embryonic lethal mutants, *spns2* ^17^ and *oep* ^18–20^, after transplantation into the *rag1* mutant.

**Figure 5:**
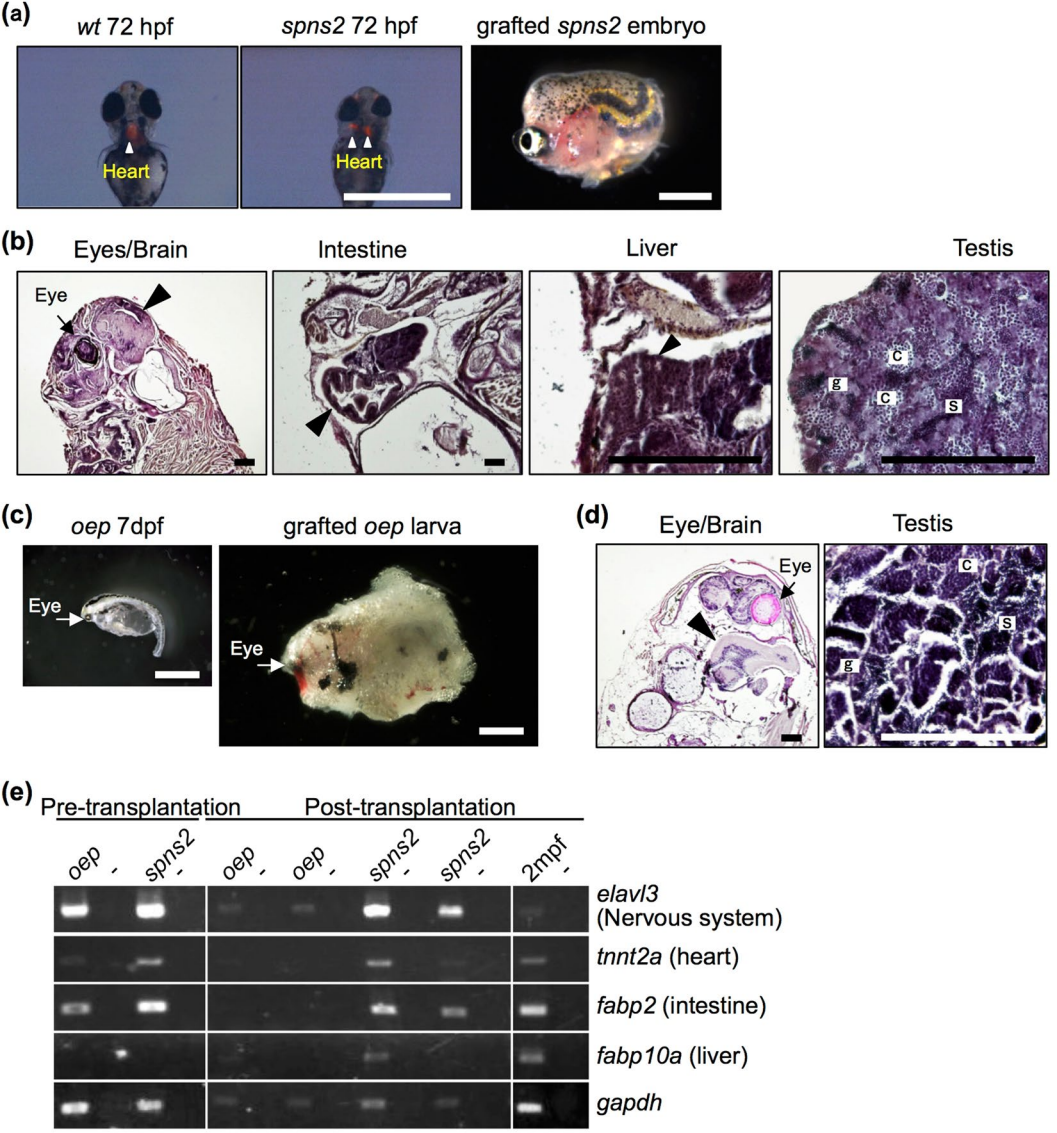
Development of lethal mutant embryos and larvae grafted into *rag1* mutants. (**a**,**c**) Morphology of 72 hpf *spns2* mutant embryos (**a**) and 7 dpf *oep* mutant larvae (**c**) before and after transplantation into germ cell-depleted *rag1* mutants. The *spns2* mutant has cardia bifida (two hearts) as determined by *cmlc2*-driven mRFP expression (arrowheads), and the *oep* mutant exhibits a single eye (arrows). Scale bar: 1 mm. (**b**,**d**) Histology of organs found in grafted *spns2* mutant embryos (**b**) and *oep* mutant larvae (**d**) at 2 months after transplantation. Arrowheads indicate each organ as labelled above the panels. Note that testes of the grafted *spns2* and *oep* mutants contained all stages of spermatogenic cells: spermatogonia (g), spermatocytes (c), and sperm (s). The heart was not observed in grafted *spns2* mutant embryos (**b**). Scale bar: 200 µm. (**e**) RT-PCR analysis of grafted *spns2* and *oep* mutants at 2 months post-transplantation. Wild-type zebrafish at 2 mpf were used as a positive control. Negative controls lacking reverse transcriptase are in alternating lanes (−). Each experiment was conducted using two independent samples.

The *spns2* mutant displays cardia bifida (two hearts) during early embryogenesis due to mutation of the *spinster homolog 2* ^17^ and dies within several days after hatching. The *spns2* mutant grafted at 72 hpf developed for two months in the recipient (Fig. 5a). The brain, eyes, intestine, liver, and testes were observed histologically (Table 1; Fig. 5b). The testis contained all stages of spermatogenesis, including sperm. Although a primitive heart was observed in 72 hpf *spns2* mutants, further heart development was not observed. RT-PCR analysis supported the existence of all organs except the heart, as *tnnt2a* was only weakly detected (Fig. 5e).

The *oep* mutant has severe defects in the development of the prechordal plate, endoderm, ventral neuroectoderm, liver, gut, and heart due to mutation of *teratocarcinoma-derived growth factor 1* (*tdgf1*)^18–20^ and dies by 10 dpf. Transplantation of the 7 dpf *oep* mutant, in which maldevelopment had already become apparent, enabled the development of the eyes, brain and testes but not the heart, intestine or liver at 2 months post-transplantation, as detected histologically (Table 1; Fig. 5c,d). The expression of *tnnt2a, fabp2*, and *fabp10a*, which were barely detected in the grafted *oep* mutants, supported these histological observations (Fig. 5e). By artificial insemination using testes of grafted *oep* mutants and wild-type unfertilized eggs, we obtained fertilized eggs that developed normally into adult fish (Supplementary Table S2). These results indicated that not only the generation of sperm but also the development of organs in embryos with lethal mutations may be rescued from beyond the lethal stage in the *rag1* mutant in the case that spermatogenesis and organogenesis were not severely affected by the mutation.

## Discussion

In the present study, we established a novel transplantation method for living whole embryos and larvae that can support their development, including embryonic lethal mutants, after subcutaneous grafting into *rag1* mutant recipients. Both the intrusion of host blood vessels into grafts and shared blood circulation between them were observed, which likely supports the development of grafted embryos over 2 months. Thus, this method can generate body compositions similar to that observed in heterochronic parabiosis by the simple grafting of embryos into juvenile or adult fish, which effectively allows tiny embryos to join the vasculature of the host, although the embryos are inside the host. This is different from the process of parabiotic zebrafish embryo generation by blastula fusion^21^. The survival of lethal embryos by this transplantation technique allows us to analyse any further defects in the mutant organs beyond the lethal stage.

Transplantation of embryos and larvae reveals that some primitive organs, such as liver, testes and heart, occasionally do not develop well in the subcutaneous conditions of adult hosts. In zebrafish, hepatocytes are histologically evident by 36 hpf, and the extrahepatic biliary duct is evident by 52 hpf^22^. Primordial germ cells migrate to the genital ridge by 24 hpf^23^. The heart is first visible at 24 hpf, and the heart tube is elongated by 26 hpf^24^. Therefore, the affected organs are in a rather early stage of development. The liver is a highly regenerative organ, and partial hepatectomy affects hepatocyte proliferation in parabiotic partners^2,3^, but to our knowledge, there have been no reports of a loss of liver tissue in these parabiotic conditions. Our previous study reported that grafted adult testes are maintained but not lost in isogenic zebrafish^25^. It has also been reported that rat infant hearts grafted to young and adult hosts grow autonomously^26^. The disappearance of the liver, testes and heart in the present study is not consistent with these findings and is likely due to a combination of the primitive organs and adult systemic environments.

To explore the possibility that the adult host environments affect the development of primitive organs in the grafted embryo, we focused on the testis because germ cells and testes can be removed by either knock down of *dead end* ^16^ or castration. Removal of host germ cells stimulated testis development in the grafted embryo. In our previous study, no adult testes disappear after transplantation even though germ cells were not removed from the host^25^. Therefore, this result indicates that the primitive testis of an embryo at 24 hpf–5 dpf has a period in which it is susceptible to the systemic environment produced by the germ cells of adult hosts during development. It is well known that the growth and development of germ cells are controlled by the pituitary-gonad endocrine system. The present study indicates that the extra pituitary and testicular somatic cells of the host stimulate the development of the primitive testis in the graft, whereas extra germ cells in the host suppress it. By comparing the molecules that appeared at different levels between these conditions, the factors originating from the germ cells, which are assumed to circulate in blood, could be identified.

The liver and heart in grafted embryos also occasionally disappeared 2 months after transplantation, although we were not able to determine the cell or organ of the host that affected them in the present study. It is possible that similar to testes, these organs also have periods of susceptibility to the age-related systemic environment of adult hosts. Specific cell types that express foreign nitroreductase can be removed by chemical ablation in zebrafish^27^. Using this technique in our whole living embryo/larvae transplantation could identify the means by which the development of the primitive liver and heart is prevented. It is assumed that each factor preventing these organs’ development differs from the factor preventing testis development because the liver and heart also occasionally disappeared during transplantation into a germ-cell-removed host.

We further observed that the sizes of the eyes and intestine at 72 dpf, similar to the liver and testes, became smaller than those of normally developing individuals. The optic lobe can be first distinguished at the 6 somite stage (12 hpf), and by the end of somitogenesis (24 hpf), the lens is spherical and has detached from the epidermis^28^. Endodermal precursors originate from marginal blastomeres, which involute early in gastrulation (5.25 hpf), and the developing gut is a cord of radially aligned cells at 36 hpf^22^. These organs may have more severe damage when embryos earlier than 24 hpf are transplanted into adult hosts. Conversely, the subcutaneous condition reduces the light stimulation of eyes and requires no digestion in the intestine of the grafts. Since the eyes of cave fish become smaller and regress^29^, a reduction of functional needs may cause the small sizes of the eyes and intestine of the grafted embryos. It is possible to determine whether the eye size is affected by darkness or extra eyes by transplantation to transparent hosts^30^ or mutants lacking eyes. Further studies utilizing specific cell ablation methods would also help elucidate if the cells or organs in the host affect development.

Recent studies reported that an aged systemic environment influences the regeneration potential of young stem cells during heterochronic parabiosis and that blood age affects the regeneration of all three germ layer derivatives during rodent blood exchange^4–6,31^. The present study reveals that the development of primitive organs, particularly of the testes and possibly the heart and liver, are affected by the aged systemic environments of the adult hosts, which originates from adult germ cells in the case of testes. Identifying the factors and molecular mechanisms that affect primitive organ development could facilitate our understanding the unique phenomenon in which the organ development is coordinated with age-related systemic environments.

## Methods

### Zebrafish

*rag1* mutant (*rag1* ^*t26683*^), *one-eyed pinhead* mutant (*oep*, *tdgf1* ^*tz257*^), and *spinster homolog 2* mutant (*spns2* ^*ko157*^) zebrafish were provided by Dr Lora Petrie-Hanson, Dr Masahiko Hibi and Dr Atsuo Kawahara, respectively. For wild-type fish, we used India or AB lines. The use of these animals for experimental purposes was approved by the committee on laboratory animal care and use at the National Institute of Genetics (approval identification numbers 27-12 and 28-13), and the experiments were conducted in accordance with the guidelines of the National Institute of Genetics.

### Transplantation of embryos and larvae

Transplantation of embryos and larvae was performed using the immunodeficient *rag1* mutant as described previously^9,32^. *rag1* mutant adult males were not fed on the day prior to transplantation. Recipient fish were anesthetized with 0.01% ethyl p-aminobenzoate (Wako, Osaka, Japan), and an incision of approximately 10 mm was made into their abdominal skin with a razor blade. Next, the muscle and skin were separated by inserting a forceps tip between the muscle and skin through this wound. The embryo or larva was then inserted into the space. To facilitate healing, the recipient fish were maintained for four days in 0.4x phosphate buffered saline (PBS) containing 10 µg/ml gentamicin (Life Technologies) without suturing the wound in the dark. Fish were subsequently reared normally and, after an appropriate period, were anesthetized to enable removal of the graft. The removed graft was fixed overnight at room temperature using 4% paraformaldehyde (PFA) in PBS or Bouin’s fixative, embedded in paraffin, and then sectioned at 5–15 µm for histological analysis.

Castrated recipients were produced by removing the testes after anaesthesia and abdomen incision with a razor blade. After fixing the wound softly by hands without suturing, fish were maintained for four days in 0.4x PBS containing 10 µg/ml gentamicin in the dark. After two weeks, fish were used for grafting the embryos/larvae. Germ cell-depleted recipients were produced by an injection of morpholinos targeting the *dead end* gene into 1–2 cell stage zebrafish embryos as described^16^.

### Microinjection of FITC-dextran solution into the heart of a living recipient

For microinjection into recipients’ hearts, we used recipient fish into which 72 hpf embryos were transplanted within 3 months after transplantation. Recipients were anesthetized, and the thorax was cut with a razor blade to expose the recipient’s heart. A total of 1.5 µl of solution containing 5% fluorescein isothiocyanate (FITC)-conjugated dextran (Molecular Probes) was placed into a glass needle using a Nanoject II auto-nanoliter injector (Drummond) and then injected into the exposed heart of the recipient. Injected recipients were transferred to standard tank water and then used for observation of blood vessels at 3 and 15 minutes after injection. The observation of blood vessel fluorescence was performed using a stereomicroscope (Leica).

### X-ray micro-computed tomography analysis

The µ-CT (Scan X mate E090S Scanner, Comscantechno) was used to collect tomography images of grafted 72 hpf embryos at 3 months after transplantation. Samples were fixed with 4% PFA in PBS overnight at room temperature. For bone scanning, the µ-CT was operated at a tube voltage peak of 60 kV and a tube current of 100 µA. Samples were rotated 360° in steps of 0.24°, which generated 1500 projection images of 992 × 992 pixels. The µ-CT data were reconstructed at an isotropic resolution of 8.9 × 8.9 × 8.9 µm. Three-dimensional tomographic images were obtained using OsiriX (www.Osirix-viewer.com) software. For scanning of soft tissue, samples were dehydrated in an ascending ethanol series (70%, 80%, 90%, 95%, 2 × 100%, 1 hr each except overnight for the last step) to remove lipids. After dehydration, samples were rehydrated in a descending ethanol series and PBS (90%, 80%, 70%, PBS, 1 hr each) and then soaked in contrast agent, a 1:3 mixture of Lugol’s solution and deionized distilled water, as previously described^33^. The µ-CT was operated at a tube voltage peak of 90 kV and a tube current of 85 µA. Samples were rotated 360° in steps of 0.144°, generating 2500 projection images of 992 × 992 pixels. The µ-CT data were reconstructed at an isotropic resolution of 6.5 × 6.5 × 6.5 µm. Three-dimensional, tomographic images were obtained using the OsiriX (www.Osirix-viewer.com) and TRI/3D-BON (RATOC Systems) software.

### RT-PCR

Removed grafts and 2 mpf fish were minced for RNA extraction, and total RNA was isolated using RNAiso Plus (Takara). cDNA was synthesized using Primescript Reverse Transcriptase (Takara). Controls in which no reverse transcriptase was added were run in parallel in the RT-PCR experiments. PCR was performed using GoTaq Master Mix (Promega). The thermal cycling conditions were as follows: an initial hold of 2 min at 95 °C followed by 35–50 cycles of 30 s denaturation at 95 °C, 30 s primer annealing at different temperatures depending on the primer pairs, and 20 s of elongation at 72 °C. The sense and antisense primer pairs were ACCCAGCTCTACCAGACAGC and TGGTTATGGGGGAGAATCTG for *ELAV-like neuron-specific RNA binding protein* (*elavl3*)^10^, CAACGAAGAAGTGGAAGAGTACGAG and TTCTCCATCGTGTTCCTGAGTG for *troponin T type 2a* (*tnnt2a*)^11^, TCATCATGACCTTCAACGGGACCT and ATTTCCAGTGTGCGGAAAGTGCTG for *fatty acid binding protein 2* (*fabp2*)^12^, CAAGAAGCTCAAGTGCATCG and TTTAAATAGAGTGATGGTGAAACG for *fatty acid binding protein 10a* (*fabp10a*)^13^, and TTCTCACAAACGAGGACACAA and CAAGGTCAATGAATGGGTCA for *glyceraldehyde-3-phosphate dehydrogenase* (*gapdh*).

### Artificial insemination

A single grafted testis was minced and ground using a microtube pestle in Hank’s saline (0.137 M NaCl, 5.4 mM KCl, 0.25 mM Na_2_HPO_4_, 0.44 mM KH_2_PO_4_, 1.3 mM CaCl_2_, 1.0 mM MgSO_4_, and 4.2 mM NaHCO_3_) and then stored on ice. Unfertilized eggs from a single wild-type female were collected onto a dish according to the method of Westerfield^34^. The suspension was added, and the dish was shaken gently for 2 minutes to mix. PBS (100 μl) was added gradually, with shaking. After an additional 2 minutes, 5 ml of fish water was added gradually. The success of fertilization was assessed at 3–5 hpf.

### Determination of organ size

Grafted 72 hpf embryos at 2 months post-transplantation, 72 hpf embryos and 2 mpf males were fixed with Bouin’s fixative overnight at room temperature and sectioned serially in paraffin at 10–15 µm thickness for histological analysis. Serial sections were stained with haematoxylin and eosin. After imaging all of the serial sections using a digital microscope camera (Keyence), the area of each organ was measured using ImageJ v. 1.48^35^. Organ volume was calculated by multiplying the section thickness by the sum of the organ area.

### Statistical analysis

Data were presented as the mean ± standard deviation of at least three independent experiments. Statistical difference between two groups was determined using Student’s *t*-test. *P* < 0.05 was considered statistically significant.

## Electronic supplementary material

Supplementary Tables and Supplementary Figures.

Supplementary Video S1: Video of grafted 72 hpf embryo.

Supplementary Video S2: µ-CT imaging of grafted 72 hpf embryo.
