# Supplementary material for: Development and growth of organs in living whole embryo and larval grafts in zebrafish
Source: Sci Rep. 2017 Nov 28;7:16508. doi: 10.1038/s41598-017-16642-5 (PMC5705650; doi:10.1038/s41598-017-16642-5)
Supplement: Supplementary file 1 — Supplementary Tables and Supplementary Figures. [file 41598_2017_16642_MOESM1_ESM.docx]

Development and growth of organs in living whole embryo and larval grafts in zebrafish

Toshihiro Kawasaki, Akiteru Maeno, Toshihiko Shiroishi & Noriyoshi Sakai

**Supplementary Tables and Supplementary Figures.**

**Supplementary Table S1.** Progression of embryogenesis in isotonic medium.

| Osmotic pressure | # of normal embryos | | | |
| --- | --- | --- | --- | --- |
|  | 0 hpf | 24 hpf | 48 hpf | 72 hpf |
| 12 mOsm | 300 | 294 | 291 | 291 |
| 300 mOsm | 300 | 270 | 258 | 258 |

**Supplementary Table S2.** Numbers of eggs successfully fertilized with sperm taken from grafted 72 hpf embryos, 5 dpf larvae and 7 dpf *oep* mutants.

| Grafted embryo | Recipient | Total number of eggs | Number of fertilized eggs | Number of hatched embryos |
| --- | --- | --- | --- | --- |
| 72 hpf | Germ cell-depleted *rag1* | 252 | 124 | 119 |
|  |  | 192 | 17 | 17 |
|  |  | 79 | 0 | 0 |
| 5 dpf | *rag1* | 204 | 158 | 158 |
|  |  | 259 | 27 | 27 |
|  |  | 178 | 81 | 81 |
| 7 dpf *oep* mutant | Germ cell-depleted *rag1* | 170 | 59 | 56 |
|  |  | 476 | 432 | 421 |
|  |  | 239 | 114 | 99 |


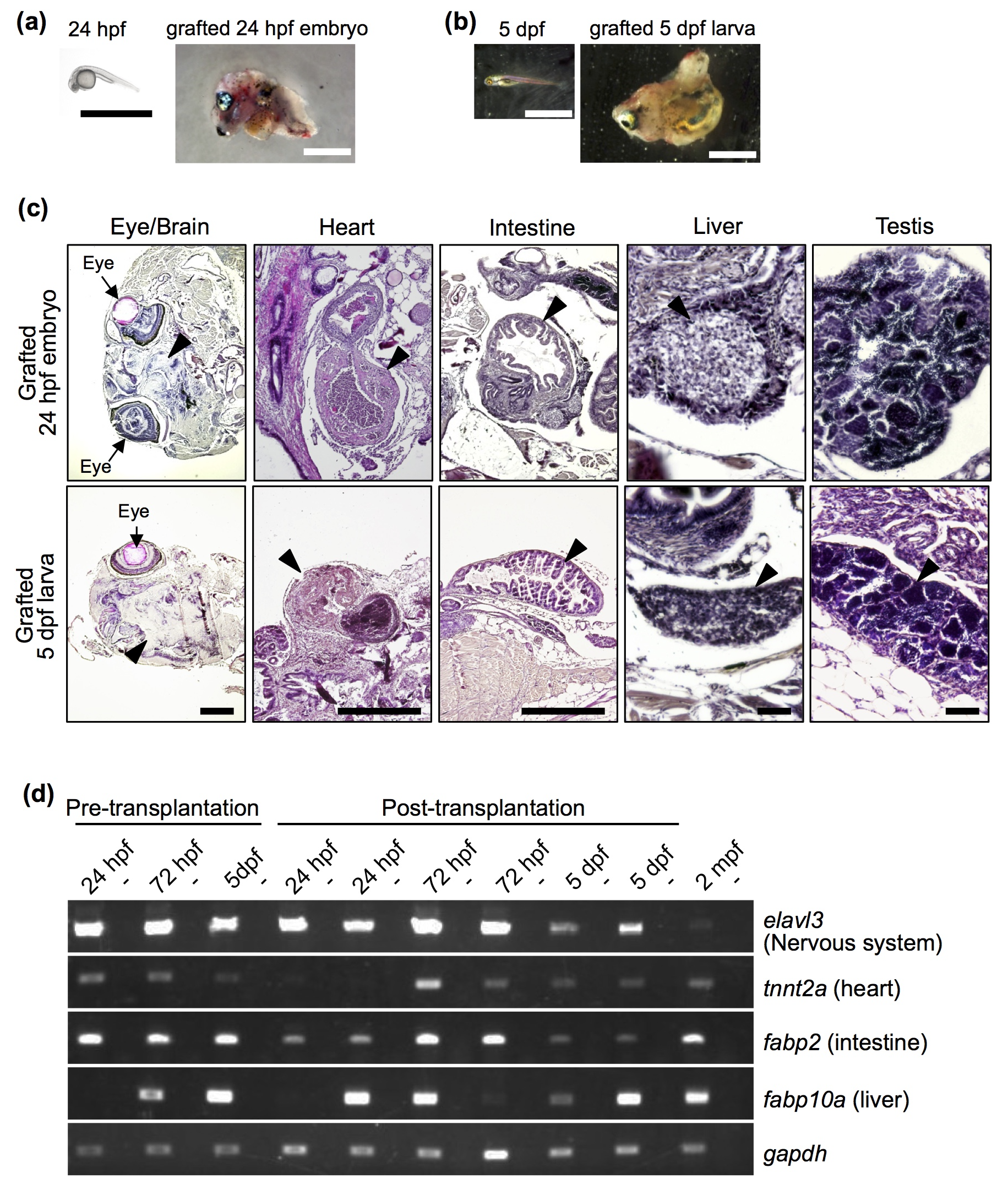


**Supplementary Fig. S1. Development of grafted 24 hpf embryos and 5 dpf larvae at 2 months post-transplantation.** (A, B) Morphology of 24 hpf embryos (A) and 5 dpf larvae (B) before and after transplantation. Scale bar: 2 mm. (C) Histology of grafted 24 hpf embryos and 5 dpf larvae. Sections were processed with haematoxylin and eosin staining, and representative major organs are shown. Note that morphologically distinct organs developed from 24 hpf embryos and 5 dpf larvae. Arrowheads indicate each organ, as labelled above the panels. Photos of the heart, liver and testis were from grafts in which those organs developed. Scale bars: 500 µm (brain, heart and intestine) and 50 µm (liver and testis). (D) RT-PCR analysis of organ-specific genes in grafted 24 hpf and 72 hpf embryos and 5 dpf larvae at 2 months post-transplantation. Duplicate experiments were performed with independent samples. Wild-type zebrafish at 2 mpf was used as a positive control. Negative controls lacking reverse transcriptase are shown in alternating lanes (-). *gapdh* was used as a positive control.

**
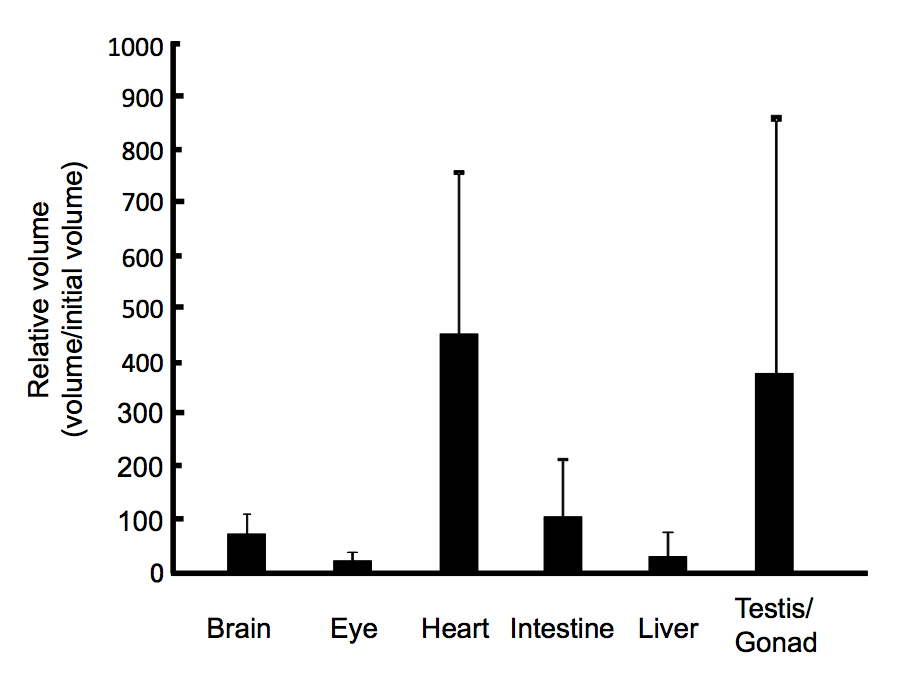
Supplementary Fig. S2. Growth of organs in grafted 72 hpf embryos.** 72 hpf embryos were subcutaneously transplanted into *rag1^t26683^* mutants, and recipients were maintained for two months. Grafts and 72 hpf embryos were serially sectioned and stained with HE. The volume of each organ was calculated from serial sections of the grafted embryos whose organs developed (n=12 brains, 11 eyes, 11 hearts, 12 intestines, 8 livers, and 6 testes) and 2 mpf males (n=3). The relative ratio of the organ size of the grafts to that of the 72 hpf embryos is presented. Note that all detected organs grew in the grafted embryos. Error bars indicate standard deviation.

**
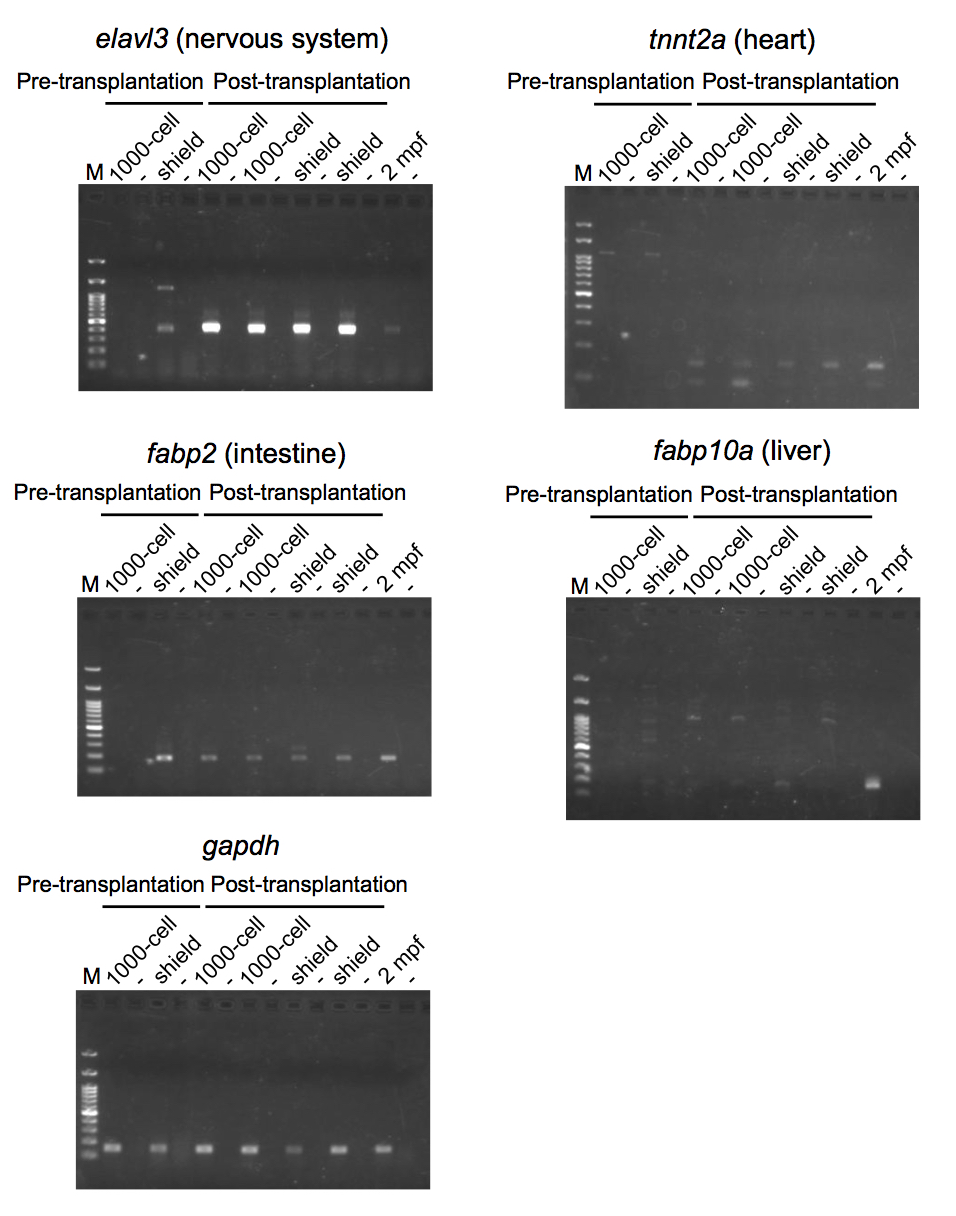
Supplementary Fig. S3. Raw electrophoresis images of RT-PCR analysis of the grafted 1000-cell stage and shield stage embryos as presented in Figure 3.** M: 100 bp DNA ladder.

**
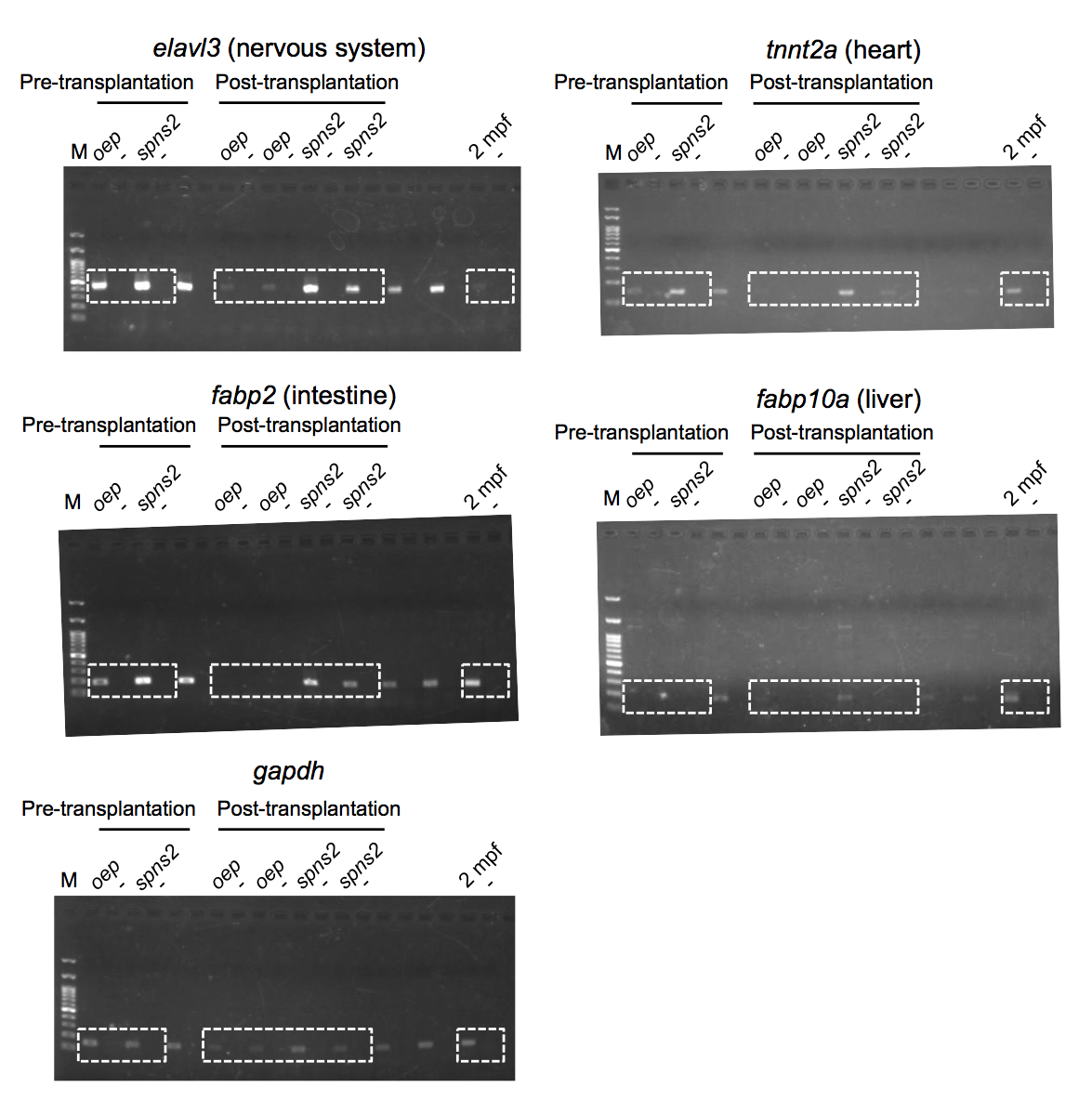
Supplementary Fig. S4. Raw electrophoresis images of RT-PCR analysis of grafted lethal mutant embryos (*spns2*) and larvae (*oep*), as presented in Figure 5.** The region enclosed by dotted lines indicates the area used in Figure 5. M: 100 bp DNA ladder.

**Supplementary Video S1: Video of grafted 72 hpf embryo.**

This video shows the beating heart of a grafted embryo in a recipient at 2 months post-transplantation, indicating that the organ grown in the recipient functions as a heart.

**Supplementary Video S2:** **µ-CT imaging of grafted 72 hpf embryo.**

This video shows a 3D image of osseous tissue of the grafted embryo. The osseous tissue of the recipient was removed using OsiriX software.
